# Two-Gene Phylogeny of Bright-Spored Myxomycetes (Slime Moulds, Superorder Lucisporidia)

**DOI:** 10.1371/journal.pone.0062586

**Published:** 2013-05-07

**Authors:** Anna Maria Fiore-Donno, Fionn Clissmann, Marianne Meyer, Martin Schnittler, Thomas Cavalier-Smith

**Affiliations:** 1 Zoology Department, University of Oxford, Oxford, United Kingdom; 2 Institute of Botany and Landscape Ecology, University of Greifswald, Greifswald, Germany; 3 Le Bayet, Rognaix, France; Institut Pasteur, France

## Abstract

Myxomycetes, or plasmodial slime-moulds, are one of the largest groups in phylum Amoebozoa. Nonetheless, only ∼10% are in the database for the small subunit (SSU) ribosomal RNA gene, the most widely used gene for phylogenetics and barcoding. Most sequences belong to dark-spored Myxomycetes (order Fuscisporida); the 318 species of superorder Lucisporidia (bright-spored) are represented by only eleven genuine sequences. To compensate for this, we provide 66 new sequences, 37 SSU rRNA and 29 elongation factor 1-alpha (EF-1α), for 82% of the genera of Lucisporidia. Phylogenetic analyses of single- and two-gene alignments produce congruent topologies and reveal both morphological characters that have been overemphasised and those that have been overlooked in past classifications. Both classical orders, Liceida and Trichiida, and several families and genera are para/polyphyletic; some previously unrecognised clades emerge. We discuss possible evolutionary pathways. Our study fills a gap in the phylogeny of Amoebozoa and provides an extensive SSU rRNA sequence reference database for environmental sampling and barcoding. We report a new group I intron insertion site for Myxomycetes in one *Licea*.

## Introduction

Myxomycetes, or plasmodial slime-moulds, are exceptional in several respects. Most striking is their life cycle: a giant multinucleate amoeba (up to several dm^2^) is formed by fusion of two amoebae. This cycle is often sexual, culminating in the formation of mainly macroscopic fruiting bodies, of astonishing variety in shape and colour, that will ultimately release billions of spores - the life cycle is illustrated in many text books (see, among others, [1]). The evolutionary success of Myxomycetes is indicated by their numbering 941 species (listed in “An online nomenclatural information system of Eumycetozoa”, http://eumycetozoa.com/data/index.php, updated 20.7.12), thus with arcellindid testate amoebae being one of the two largest groups within the phylum Amoebozoa. Myxomycetes are a firmly established clade within Amoebozoa, probably sisters to Dictyostelea, and more distantly related to Protostelida, Variosea, and perhaps Archamoebae, but relationships amongst these five taxa remain uncertain [2]–[7].

Myxomycetes are divided into two subclasses Exosporeae Rostaf. (i.e. *Ceratiomyxa*) and Myxogastria Fr. [8]. The primary phylogenetic bifurcation within Myxogastria is between the dark-spored and bright-spored clades [2], [9], superorders Columellidia and Lucisporidia respectively [8]. Lucisporidia has 34% of myxomycete species and is generally divided into two orders (Liceida Jahn, 1928 and Trichiida Macbride, 1922), 22 genera and 318 species (http://eumycetozoa.com/data/index.php, updated 20.7.12) (Table 1).

**Table 1:** Systematic treatment of the class Myxomycetes (according to [12]), number of genera and species (according to Nomenmyx, http://eumycetozoa.com/data/index.php, updated 20.7.12) and percentage of genera and species sequenced in this study.

			Genera/			Species/	# Sequences obtained	
Order	Family	Authors	Family	Genus	Authors	Genus	Genera	Species
**Liceida**	Cribrariidae	Corda	2	*Cribraria*	Pers.	46	1	2
				*Lindbladia*	Fr.	1	1	1
	Dictydiaethaliidae	Nann.-Bremek. ex H.Neubert, Nowotny& K. Baumann	1	*Dictydiaethalium*	Rost.	2	1	2
	Liceidae	Chevall.	1	*Licea*	Schrad.	70	1	4
	Listerellidae	E. Jahn ex H.Neubert, Nowotny& K. Baumann	1	*Listerella*	E. Jahn	1	0	0
	Reticulariidae	Chevall.	3	*Lycogala*	Adans.	6	1	1
				*Reticularia*	Bull.	11	1	2
				*Tubifera*	JF.Gmel.	7	1	1
	**Total Liceida**		**8 Genera**			**144**	**7 (87.5%)**	**13 (9.0%)**
**Trichiida**	Arcyriidae	Rostaf. ex Cooke	5	*Arcyodes*	OF.Cook	1	1	1
				*Arcyria*	FH.Wigg	49	1	3
				*Arcyriatella*	Hochg.& Gottsb.	1	0	0
				*Cornuvia*	Rostaf.	1	1	1
				*Perichaena*	Fr.	29	1	3
	Dianemidae	T. Macbr.	2	*Calomyxa*	Nieuwl.	2	1	1
				*Dianema*	Rex	12	1	2
	Minakatellidae	Nann.-Bremek. ex H.Neubert, Nowotny& K. Baumann	1	*Minakatella*	G. Lister	1	0	0
	Trichiidae	Chevalier	6	*Calonema*	Morgan	5	0	0
				*Hemitrichia*	Rostaf.	26	1	2
				*Metatrichia*	Ing	6	1	2
				*Oligonema*	Rostaf.	7	1	2
				*Prototrichia*	Rostaf.	1	1	1
				*Trichia*	Haller	33	1	4
	**Total Trichiida**		**14 Genera**			**174**	**11 (78.6%)**	**22 (12.6%)**
	**Total Lucisporidia**		**22 Genera**			**318**	**18 (81.8%)**	**35 (11%)**

**Figure 1:**
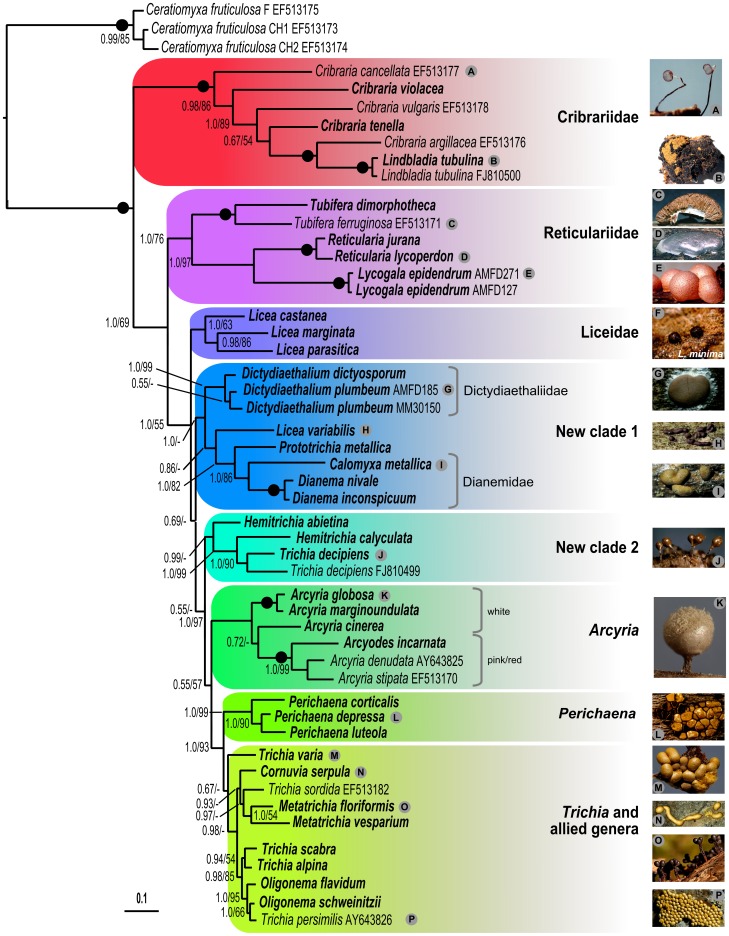
SSU rRNA gene tree of Lucisporidia derived by Bayesian inference of 1325 nucleotide positions of 51 sequences, with *Ceratiomyxa fruticulosa* as outgroup. Species names are followed by GenBank accession number, except for sequences obtained during this study (in bold), whose accession numbers and collection sites are in Table S1. Clades are highlighted and labelled according to current classification or as new. Bayesian posterior probabilities (BPP)/ML bootstrap replicates (MLB) are shown for each branch; dashes indicate a conflicting topology in the ML tree; a dot on the line indicates maximum support in both analyses. The scale bar indicates the fraction of substitutions per site. Credit photos: A, F, G, J–M: Michel Poulain; B–E, H, I, N–P: Alain Michaud.

Readers unfamiliar with these taxa can find excellent illustrated descriptions [10], [11], [12] or consult the online searchable database of the eumycetozoan project at the University of Arkansas (http://slimemold.uark.edu/databaseframe.htm, last accessed: 19 Sep. 2012). We shall use only a few specialized terms, explained below. Differentiation of the plasmodium into a fruiting body (sporophore) forms three structures: peridium, capillitium, and spores. The peridium is the wall surrounding the fruiting body, and the capillitium is a system of threads interwoven throughout the spores (best seen in Fig. 1K). The sporophore is named according to its shape: most common are individual sporocarps or sporangia, stalked (Fig. 1 A, J, K, O) or sessile (Fig. 1 F, H, I, L, N). In the former, the ensemble of the spore mass, peridium and capillitium (the two latter facultative) is called the sporotheca, to differentiate the “fertile” part from the stalk. Large sporophores (>1 cm) are mostly a compound of multiple sporangia: if the sporangia are delimited, it is called a pseudoaethalium (Fig. 1 C, G), and an aethalium when it looks like a single mass (Fig. 1 B, D, E).

Considerable taxonomic value has been placed on the capillitium: its presence/absence distinguishes the two classical orders of Lucisporidia (present in Trichiida; lacking in Liceida). Its intrinsic features - such as length, branching patterns and surface ridges, are used to characterize families and genera in Trichiida. The order Liceida (with 5 families: Table 1) comprises such a variety of forms, sizes, and shapes that it has been long considered as heterogeneous [13], [14]. It includes some of tiniest fruiting bodies, as in *Licea* (Fig. 1 F), and some of the largest, as those of the Reticulariidae (Fig. 1 C–E) and *Dictydiaethalium* (Fig. 1 G). In contrast, Trichiida appear to be more homogeneous as a whole, but distinctions between families and genera are difficult [13]. Accordingly, two (Dianemidae and Trichiidae) or three families (the same as above plus Arcyriidae) have been recognized. Similarly, the fourteen genera (Table 1) are difficult to delimit as many species possess features in common with two genera [13].

Currently, phylogenetic clarification of the position of Myxomycetes in Amoebozoa and investigation of their ecological role in soil is hampered by the lack of a sequence reference database: in the 101 small subunit (18S or SSU) ribosomal RNA gene sequences reported [15] only 11 belong to the bright-spored Lucisporidia. To compensate for this bias, and to shed light on the taxonomic conundrum of Lucisporidia, we provide 66 small subunit rRNA (SSU) and elongation factor 1α (EF-1α) gene sequences for 81.8% of the genera of Lucisporidia (Table 1). Obtaining the sequences has been extremely difficult, due to their great genetic divergence, not only from the sister-clade Columellidia but also within the group. Our phylogenetic analyses of single- and two-gene trees lead to a congruent and mostly well-supported topology, which challenges the current classification and allows us to hypothesize evolutionary pathways.

## Materials and Methods

### Specimens

All specimens were field-collected and deposited in herbaria (Table S1). To ensure a coherent approach for this taxonomically difficult group, all specimens were identified by the third author. The present study used the Zoological Code for easier comparison with the latest classification at the high-taxon level [8], while descriptions and names of families and genera were based on the most recent treatise [12], modified with the Zoological Code endings.

### DNA Extraction, Amplification and Sequencing

DNA was extracted from 5–6 adjacent sporophores (most probably arising from a single plasmodium), or from a portion of a large aethaloid fruiting-body, as previously described [16]. To obtain the highly divergent SSU sequences, specific primers had to be designed (Fig. S1). The presence of large introns, sometimes with strong secondary structure (especially intron S529) required the use of the “primer walking” method. For the EF-1α gene, in addition to already published primers [2], [9] the following primers were used (5′–3′, “R” indicates a reverse primer): at the extremities of the gene, 1FTri GGTAAGTCAACCACCACTGG, 10RTri CATATCACGGACGGCAAAACG; in the middle of the gene: E6FBright AACAAGATGGAYGACAARTC, E8RBright CCRATACCTCCRATYTTGTA. In some cases, “primer walking” had also to be used. Amplification parameters were adapted according to the elongation time (depending on the length of the expected product, 1–2 min) and annealing temperature of the primers (52–58°C). Amplicons were purified using SureClean (Bioline) or with the PCR DNA and Gel Band Purification kit (GE Healthcare Life Science), then sequenced at various facilities (Department of Genetics and Evolution, University of Geneva; Zoology Department, Oxford University; and Zoology Institute, University of Greifswald). Sequences are deposited in GenBank under accession numbers JX481280–JX481345 (Table 1).

### Alignments

GenBank nucleotide database was searched for all sequences belonging to the orders Trichiales (or Trichiida) and Liceales (or Liceida). Half the 22 SSU sequences thus retrieved appeared actually to be fungi or other contaminants (Table S2), and one, *Arcyria cinerea* AF239231, although genuine, was too short to be included. Nineteen EF-1α sequences, on the other hand, although giving coherent BLAST matches, had to be excluded for insufficient length or quality, in particular because of the presence of indels disrupting the reading frame (Table S2). The genuine, high quality Lucisporidia sequences were aligned by hand to our existing myxomycete alignments, using BioEdit version 7.0.9 [17]. The final SSU alignment comprised 48 Lucisporidia taxa, plus 3 sequences of *Ceratiomyxa fruticulosa* as outgroup, in total 51 sequences. Because of the high divergence between sequences, only 1325 unambiguously aligned positions could be retained for phylogenetic analyses, most of the variable helices had to be excluded. Even so, the alignment displayed an astonishingly high variability (0 constant and 808 parsimony-informative sites, 929 site patterns). A proportion of gaps and completely undetermined characters of 8.14% was mainly due to the partial sequences of both *Reticularia* species.

The final EF-1α alignment comprised 38 sequences of Lucisporidia plus 3 sequences of *Ceratiomyxa fruticulosa* as outgroup. All amino-acid positions could be unambiguously aligned; the final alignment comprised 380 positions showing little variation (26.1% constant and only 78 parsimony-informative sites, 203 site patterns). A proportion of gaps and completely undetermined characters of only 3.2% was mainly due to the partial sequences of *Calomyxa metallica* and *Cornuvia serpula*.

### Phylogenetic Analyses: SSU

The TIM2 model taking into account a gamma-distributed rate heterogeneity among sites and a proportion of invariable sites (I+gamma) was selected using jModelTest 0.1.1 [18] under the Akaike Information Criterion (-ln Likelihood = 24300.743; proportion of invariable sites = 0.163; gamma shape = 0.581). Accordingly, the relative substitutions rates A–C (1.37) and A–T (1.56); C–G and G–T (both 1.00) were quite similar. Maximum likelihood (ML) analyses were run using Treefinder [19] with the TIM2+I model and a 4 rate categories gamma distribution, for 100 bootstraps replicates with the default settings, to obtain a 50% consensus tree (log likelihood = −24363.08). Bayesian analyses were run using MrBayes version 3.2 [20] with the GTR model and an 8 rate category gamma distribution. The GTR model estimates two rate parameters more than TIM2 (which cannot be implemented in MrBayes), but Bayesian inference is relatively robust to over-parameterisation (see the manual at http://mrbayes.sourceforge.net/manual.php, last accessed 6.9.12). Two million generations were run, trees were sampled every 100 generations. Convergence of the two runs (Average Standard of Split Frequencies ≤0.01) was reached after 930000 generations; burnin was set accordingly leaving 10701 trees per run to be summarized (log likelihood = −24340.33, proportion of invariable sites = 0.158911, alpha = 0.575984, ESS min. value <153).

### Phylogenetic Analyses: EF-1α

The best evolutionary model for amino-acids was estimated using MrBayes for 1 million generations. The Jones model was unambiguously selected (probability = 1.0, standard deviation 0.0). Under this model, the analysis was run on the freely available Oslo Bioportal at the University of Oslo (https://www.bioportal.uio.no/, last accessed Oct. 2012) for 4 million generations; trees were sampled every 100 generations. Stationarity (Average Standard of Split Frequencies ≤0.01) was reached after 2861000 generations, and burnin set accordingly, leaving 11391 trees per run to be summarized (log likelihood = −4210.14). Maximum likelihood (ML) analyses were run using Treefinder [19] with the same model, for 100 bootstrap replicates with the default settings, to obtain a 50% consensus tree.

### Phylogenetic Analyses: Combined SSU and EF-1α

The two-gene alignment comprised 41 sequences and 1705 positions, with 1125 distinct patterns and a proportion of gaps and completely undetermined characters of only 8.11%. The same evolutionary models described above were applied on each partition. Mr Bayes was run on the Oslo Bioportal for three million generations; trees were sampled every 100 generations. Convergence of the two runs was reached after only 210000 generations, trees obtained before convergence were discarded as burnin, and the remaining 27901 trees per run were summarized (log likelihood = −25929.46, alpha SSU = 0.638744, alpha EF-1α = 0.236029). Maximum likelihood analyses were conducted using RAxML version 7.2.8 [21], with 1000 rapid bootstrapping and subsequent thorough ML search, using the two distinct models with joint branch length optimisation (log likelihood = −25974.549151). SSU and EF-1α alignments are available as Supporting Information S1, S2, S3.

## Results

### Phylogenetic Analyses

**Figure 2:**
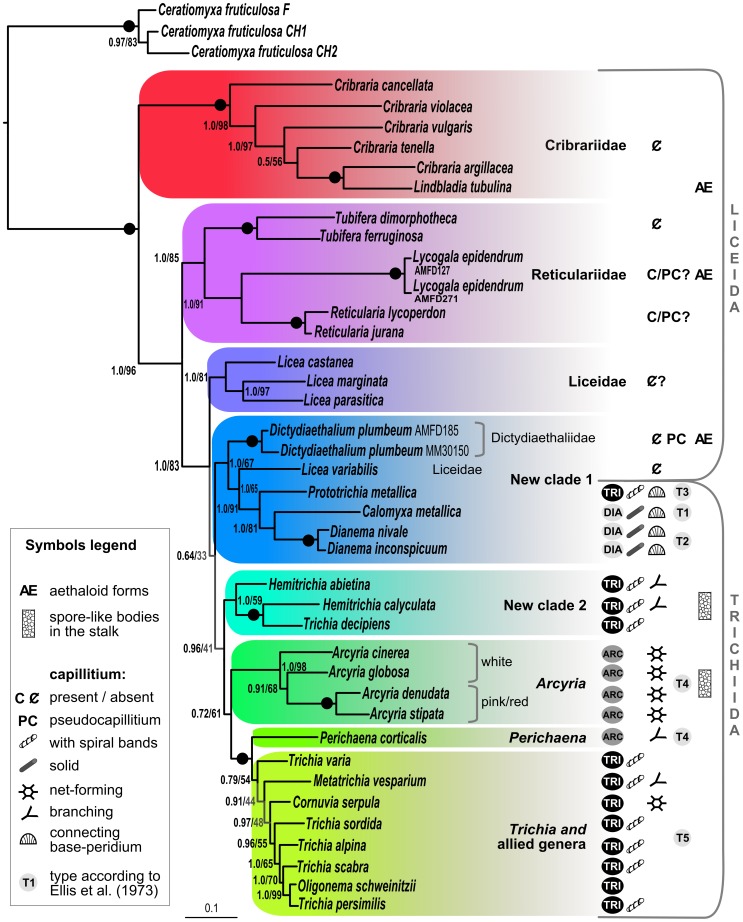
Bayesian phylogeny of Lucisporidia inferred from concatenated alignments of SSU rRNA and EF-1α genes, based on 41 sequences and 1705 positions, with *Ceratiomyxa fruticulosa* as outgroup. Clades are highlighted as in Fig. 1. Bayesian posterior probabilities (BPP)/ML bootstrap replicates (MLB) are shown for each branch; a dot on the line indicates maximum support in both analyses. In Trichiida, classical families (according to [12]) are indicated by an ellipse with the initials (Arc = Arcyriidae; Dia = Dianemidae; Tri = Trichiidae). The scale bar indicates the fraction of substitutions per site.

We obtained 66 new sequences, 37 SSU and 29 EF-1α for 35 taxa (Table S1). We assembled them with the few publicly available genuine, good-quality lucisporidian sequences in two separate alignments and a combined one. The results of our phylogenetic analyses are presented as a SSU tree (Fig. 1) and a combined SSU+EF-1α tree with fewer taxa (Fig. 2). Both trees presented the same topology, but the second had increased support for the basal branches. On the other hand, the EF-1α gene alone is too conserved to provide enough informative sites, resulting in a tree with mainly unresolved branches, provided in Fig. S2. Both SSU and two-gene trees strongly place Cribrariidae as a monophyletic lineage sister to all other Lucisporidia (Bayesian posterior probabilities (BPP) 1.0; ML bootstrap replicates (MLB) 0.98%). These are divided into seven clades, with the holophyletic Reticulariidae sister to all the others (BPP 1.0; MLB 0.85). The remaining clades are named Liceidae (pro parte) (BPP 1.0; MLB 0.81), new clade 1, new clade 2, *Arcyria*, *Perichaena* and “*Trichia* and allied genera” (Fig. 1 and Fig. 2).

### Group I Introns in the SSU

We found 37 group I introns in all nine insertion sites previously recorded for Myxomycetes [22] and in a new site S1210. Introns had a mean length of 669 bp (maximum 1557 bp), representing up to 70% of the total sequence and were present in 18 sequences. The sequence of *Licea marginata* was remarkable for hosting seven introns, including S1210 (Table S3). The analysis of the sequence of Lma.S1210 (named according to [23]) revealed all characteristics of group I introns, i.e. nine paired elements (P1–P9), the P3–P7 pseudoknot (positions 3937–41 and 4008–12 of the sequence JX481296, see also Supporting information S1), the G binding site in the P7 (pos. 3935 and 4013), and the internal guide sequence at the end of the exon matching the 10^th^ to 15^th^ nucleotides of the intron (pos. 3656–61 and 3671–76). More precisely, the extended structures in the P2, P5 and P9 segments assign the intron Lma.S1210 to subgroup IC1 [24]. In addition, it shares with other myxomycete IC1 introns the absence of base pairing between the 3′-exon and the internal guide sequence within the intron [25]: all these features make Lma.S1210 a typical group IC1 SSU myxomycete intron, in spite of its new insertion position (Fig. S3).

### Spliceosomal Introns in EF-1α

An intron that seems to be obligatory for Myxomycetes was also present in all our new 29 sequences [2], [9], [26], [27]. It lies at position 460 in the alignment provided (Supporting Information S2). Its very variable length ranges from 44 to 723 bp (in *Lycogala epidendrum* AMFD271). This insertion position is not unique to Myxomycetes [28]. Additional introns were found in three Cribrariidae: *Cribraria violacea*, *C. tenella* and *Lindbladia tubulina*.

## Discussion

### Paraphyly of the Two Orders in Lucisporidia

The combination of the two genes produces a much better supported topology than each gene separately. Our results do not support the division of Lucisporidia into the two classical orders, Liceida and Trichiida being paraphyletic; the consistently although not well-supported new clade 1 has representatives of both classical orders (Fig. 1, Fig. 2). The validity of the absence of the capillitium to define Liceida has been questioned [13], since a capillitium may be present in *Licea*, *Reticularia* and *Lycogala*. In *Licea* the processes arising from the peridium of at least three species could be a rudimentary (or vestigial) capillitium [29], and in *Lycogala epidendrum* the so-called pseudocapillitium may be in fact true capillitium. In summary, the current taxonomy is based on assumptions that are neither supported by molecular phylogeny nor by morphology. Our phylogenies instead suggest that the largest evolutionary distance lies between Cribrariidae and the remaining Lucisporidia (Fig. 1, Fig. 2).

### Early Divergence of Cribrariidae and Derived Nature of *Lindbladia*

The genus *Cribraria* Pers. stands out for its homogeneity and is distinctive in many traits, including pigments [30]. The sporophores are always stalked, except in *Cribraria argillacea*, where the stalk is short or missing (Fig. 1B). The stalked sporangium seems to represent the most ancestral condition for Myxomycetes, since it is also dominant in Echinosteliida, one of the two primary branches of Columellidia [2]. The peridium persists only as a more or less developed disc at the base of the sporotheca and otherwise as a net surrounding the spore mass (Fig. 1 A). Only six species of the family have been sequenced, and their reciprocal genetic distances are very large (long branches in Fig. 1, Fig. 2). Therefore it is not excluded that a more comprehensive sampling would alter the present picture, making taxonomic changes premature, including the elevation of the family to a higher rank. *Cribraria argillacea* and *Lindbladia tubulina* appear closely related, together forming a terminal branch of Cribrariidae (Fig. 1, Fig. 2). The monospecific genus *Lindbladia* Fr. was created to accommodate the aethaliate form of *Lindbladia tubulina*, which contrasts with the stalked sporophores of *Cribraria*. It was assumed that such distinct forms as aethalia and sporophores could not belong in the same genus [11]. *Cribraria argillacea* forms compact clusters of fruiting bodies, with or without a short stalk (an exception in this genus). *Lindbladia tubulina* shows a continuum of forms: compact clusters of fruiting bodies, with or without stalk, as in *Cribraria argillacea*, forms where the individual sporophores can hardly be seen and real, few centimetres large, pseudoaethalia (Fig. 1 B). Specimens with closely assembled sporocarps are difficult to assign to one species or the other [11]. Our results suggest that the pseudoaethalium of *Lindbladia tubulina* is a derived character in family Cribrariidae, and that *Lindbladia* may not deserve the rank of a genus.

### Multiple Origins of Aethaloid Fructifications

The large (1–10 cm) fruiting bodies called aethalia and pseudoaethalia have been suggested to have evolved by the coalescence of single fructifications [13]. Our results show that such forms are found in three distinct clades: in Cribrariidae (*Lindbladia tubulina*), in all Reticulariidae and in Dictydiaethaliidae (new clade 1) (Fig. 1, Fig. 2). In addition, they exist in all major divisions of dark-spored Myxomycetes (Fuscisporida), in Stemonitina in the “*Comatricha*” group (*Brefeldia*, *Amaurochaete*) and in both families of Physarina (*Fuligo*, *Mucilago*) (for their phylogenetic placement, see [16]). This widespread occurrence suggests multiple separate origins of aethaloid forms and convergent evolution.

**Figure 3:**
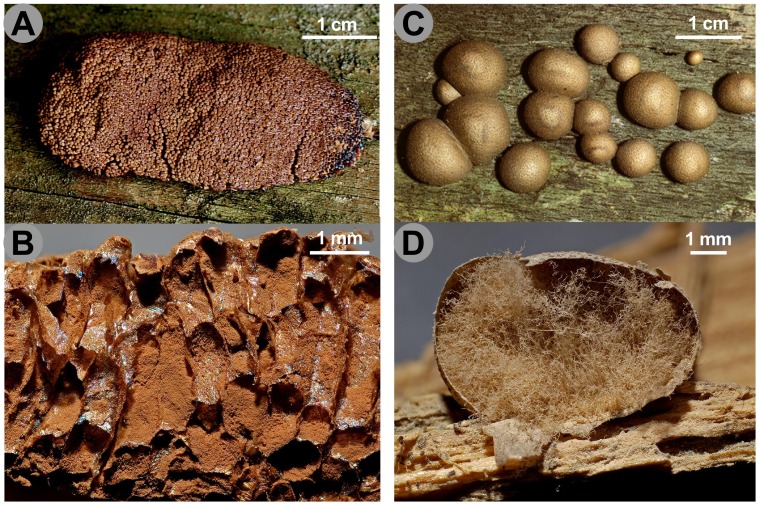
Pseudoaethalia and aethalia in Reticulariidae. **A.** *Tubifera ferruginosa*, pseudoaethalium seen from above. **B.** Vertical section showing sporangia surrounded by peridia; note the lack of capillitium in the spore mass. **C.** *Lycogala epidendrum*, aethalia seen from above. **D.** Vertical section of the same, with the spore mass blown away and the abundant pseudocapillitium. Scales and colours are approximate. Credit photos: Michel Poulain.

In Reticulariidae an interesting pattern, distinguishing pseudoaethalia from aethalia is observed. In one clade, consisting of *Tubifera ferruginosa* and *T. dimorphotheca*, the fruiting bodies are composed of closely compressed sporangia, retaining their peridium (pseudoaethalia) (Fig. 3 A, B). In the other clade, true aethalia are formed, as in *Reticularia jurana*, *R. lycoperdon* and *Lycogala epidendrum*, with fruiting body appearing as a large, single mass, where the individual sporangia cannot be distinguished (Fig. 3 C, D).

### Ontogeny and Evolution of the Capillitium

From the few studies on the ontogeny of the capillitium, at least two main patterns may be deduced: the capillitium can be laid down in anastomosing vacuoles (see, among others [31], [32], [33]) or in invaginations of the plasma membrane [34], [35], [36]. In the first process, which we call “vacuolar capillitium”, the material that will form the capillitium is deposited at a very early stage of fruiting body development by incoming vesicles in vacuoles that anastomose to form a long row [32], [35]. In the other form of capillitium development, which we call “peridial capillitium”, capillitial threads are formed in connection with the developing peridium, by invaginations of the plasma membrane, and will stay connected with the peridium [37], [38]. Both types of capillitium are found in classical Trichiida [33], [35] and in Physarida [34], [36]. Thus, both ways of capillitium deposition have been observed in Lucisporidia and in Fuscisporida. However, it is very hard to decide whether a capillitium was present ancestrally in Myxomycetes and was lost multiple times or whether it evolved convergently at least twice, in Lucisporidia and in Fuscisporida, or more times. If further ultrastructural studies revealed an ancestral origin, then in Lucisporidia the capillitium would have been lost without doubt in Cribrariidae, in *Dictydiaethalium, Tubifera* and in most *Licea* species. However, in *Dictydiaethalium* and *Tubifera*, *Reticularia* and *Lycogala*, filaments of probably diverse origins are referred to as “pseudocapillitium”.

### The Identity of New Clade I and the “Pseudocapillitium”

**Figure 4:**
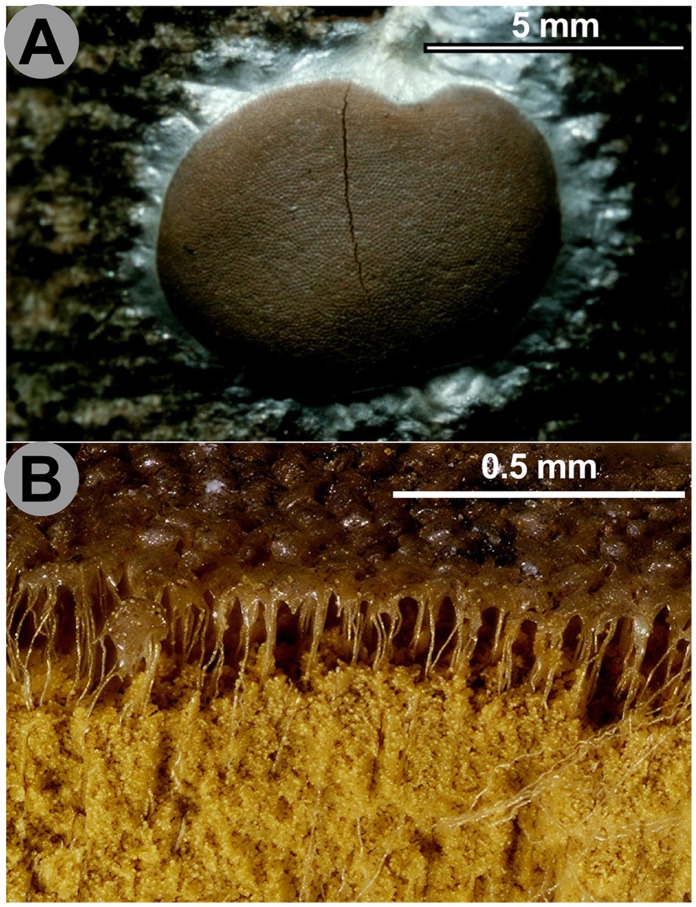
Pseudoaethalium of *Dictydiaethalium plumbeum*. **A.** **** Pseudoaethalium seen from above. **B.** Vertical section with the spore mass partially blown away, showing the renmants of the peridia as hexagonal caps on the upper surface and vertical fine threads connecting them to the base of the fructification. Scales and colours are approximate. Credit photos: Michel Poulain.

The term “pseudocapillitium” refers to filaments found in the aethaloid fructifications in Reticulariidae and Dictydiaethaliidae, regarded as the remnant of sporangial walls. From our results and from a critical analysis of morphological and ultrastructural past observations, it appears that distinct processes are jumbled under the name “capillitium”, while the term “pseudocapillitium” may be redundant. In Reticulariidae, very different types of pseudocapillitium are found: in *Lycogala*, it is composed of hollow, branched tubes arising from the inner surface of the peridium [39] (Fig. 3 D); in *Reticularia*, it is a tridimensional network of more or less flattened structures. In the non-related Dictydiaethaliidae, the peridium persists at the top of the tightly compressed sporangia as a hexagonal plate, while the peridia between sporangia remain only as fine threads connecting the angles of the plate to the base of the pseudoaethalium (Fig. 4 A, B). TEM studies on capillitial ontogeny are needed to assess to what degree the structures referred to as pseudocapillitium in *Reticularia, Lycogala* and Dictydiaethaliidae are homologous, and how they are related with the different types of “true” capillitium.

**Figure 5:**
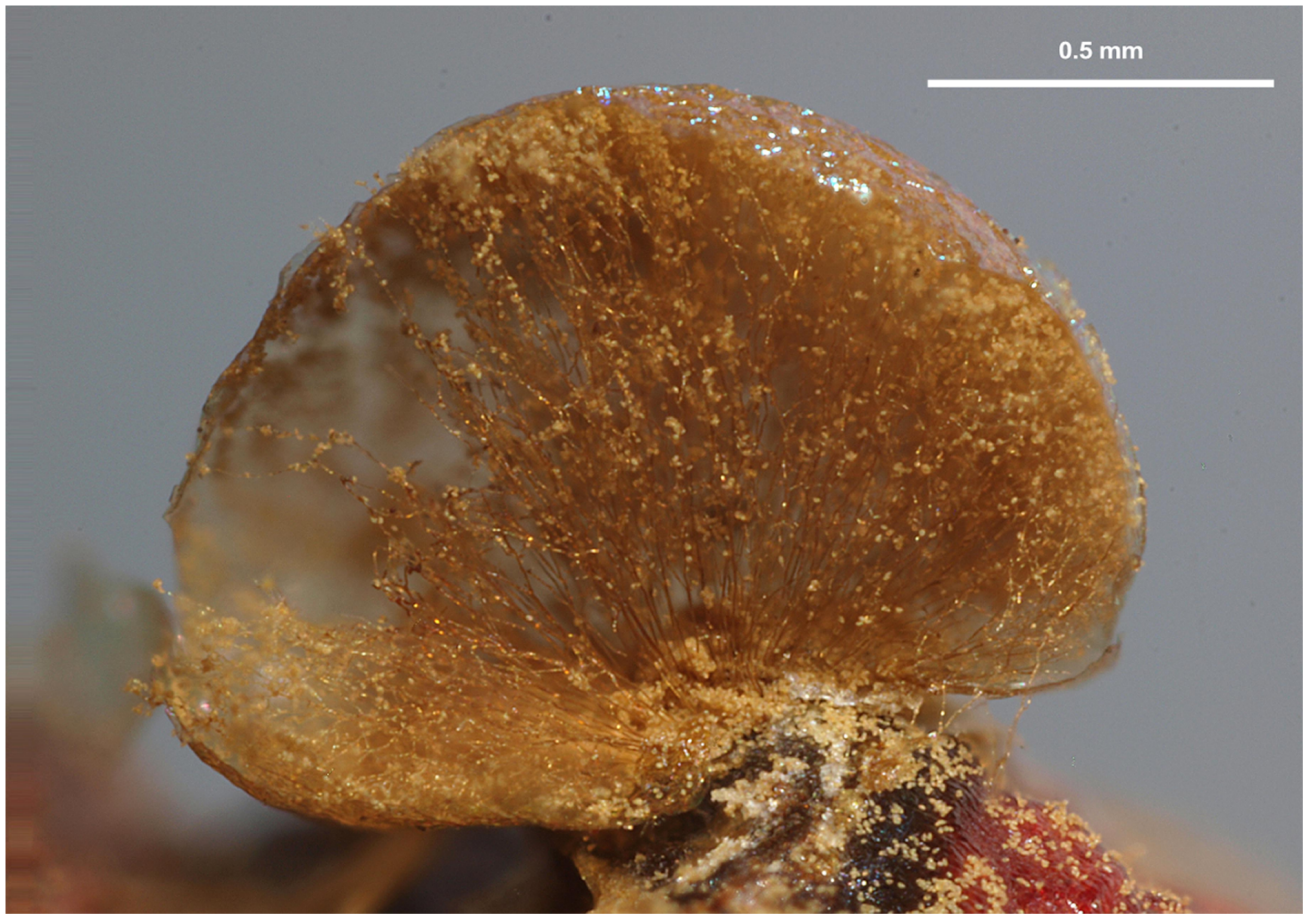
*Dianema nivale*, vertical section with the spore mass partially blown away, showing the capillitium connecting the peridium with the base of the fructification. Scale and colours are approximate. Credit photos: Michel Poulain.

In new clade 1, Dictydiaethaliidae are associated with Dianemidae, *Prototrichia metallica* (with hollow capillitium) and *Licea variabilis* (lacking capillitium). A capillitium connecting the peridium to the base of the sporotheca is a characteristic common to Dianemidae and *Prototrichia metallica* (Fig. 5). Interestingly, a scanning electron microscopic study of several Trichiida recognized five groups of capillitium, the first three of which are found in new clade 1: *Calomyxa metallica* (Type I), *Dianema* (Type II), *Prototricha metallica* (Type III) [40]. This pattern supports the phylogenetic relationships of these species within new clade I (Fig. 1, Fig. 2). The question arises whether the capillitial Type I, II and III could be homologuous to the “peridial threads” of *Dictydiaethalium*. Should this be true, the fruiting bodies of *Licea variabilis, Prototrichia metallica, Calomyxa metallica* and *Dianema* spp. would be reduced aethalia (Fig. 2).

### *Licea* and the “Protoplasmodium”

*Licea* species share a common feature with Echinosteliida, that a single sporophore arises from a very small plasmodium: this tiny type of plasmodium has been considered primitive, and therefore named protoplasmodium [41], although it has been shown that, in *Echinostelium,* the plasmodium divides into small units before fruiting [42]. The “protoplasmodium” assumption is challenged by recent results: *Ceratiomyxa fruticulosa,* which forms large plasmodia [43], is sister to Myxogastria [2], [8]. This lends support to “protoplasmodia” being derived and probably of independent origins in *Licea* and *Echinostelium*. In our tree, *Licea* would be monophyletic if *L. variabilis* were excluded. It has been suggested that *Licea variabilis* (Fig. 1 H), with fruiting bodies much larger (up to 1cm) than a typical *Licea*, should have been assigned to *Perichaena* [13], where a capillitium is sometimes absent. Our trees support the idea that *Licea variabilis* is misclassified, but not that it is a *Perichaena*. Instead we show here that *Licea variabilis* belongs to new clade 1, composed of species of Liceida and Trichiida (Fig. 1, Fig. 2). It should perhaps be placed in a new genus, possibly within a broadened Dianemidae, though this conclusion is tentative as our sampling still does not adequately reflect the variability of *Licea*, a large and heterogeneous genus, and the basal branching of clade 1 is only weakly supported.

### Families of Trichiida: Arcyriidae and Trichiidae

**Figure 6:**
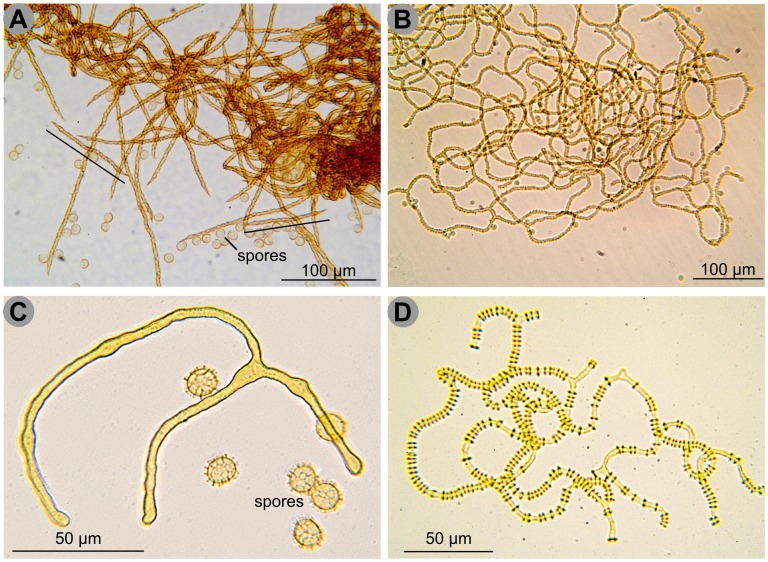
Four different capillitial threads in Trichiida. **A.** Capillitial threads of *Trichia varia*: isolated threads sculptured with spiral bands, two very short ones are indicated by a black line. **B.** Capillitial threads of *Arcyria obvelata,* forming a network and sculptured with spines. **C.** Capillitial threads of *Oligonema flavidum*, short and in this case branched, smooth. Note the reticulate ornamentation of the spores, similar to that of *Oligonema schweinitzii* and *Trichia persimilis*. **D.** Capillitial threads of *Cornuvia serpula*, branched and ornamented with rings. Scale and colours are approximate. Credit photos: Michel Poulain.

There is no general agreement on the delimitation of Arcyriidae and Trichiidae: they have been separated on the basis of the non-birefringence of the capillitium under polarized light [11], [44], a character discarded by some authors, e.g. Lado et al. [10]. Nonetheless, it is generally accepted that in Trichiidae the capillitium is mostly made of isolated threads sculptured with spiral bands (Fig. 6 A), while in Arcyriidae it is mostly net-forming and smooth or variously sculptured with warts, rings or spines, but not with clear spiral bands [10], [12] (Fig. 6 B). This classification is challenged by the genus *Metatrichia,* possessing a branching capillitium with spiral bands, which has been alternatively placed in Arcyriidae or in Trichiidae [10], [11], [12]. Species with capillitium made of isolated threads but without spiral bands, as *Oligonema* (Fig. 6 C) or with *Arcyria*-like rings, as *Cornuvia* (Fig. 6 D), have nevertheless been included in Trichiidae. Our phylogenetic results do not support the existing demarcation between these two families; instead we see the emergence of several clades: new clade 2, *Arcyria*, *Perichaena* and “*Trichia* and allied genera”. Their mutual relationships are not well-supported, except that *Perichaena* is robustly sister to “*Trichia* and allied genera”, which contradicts its current placement in Arcyriidae.

### New Genera and Redundant Ones

**Figure 7:**
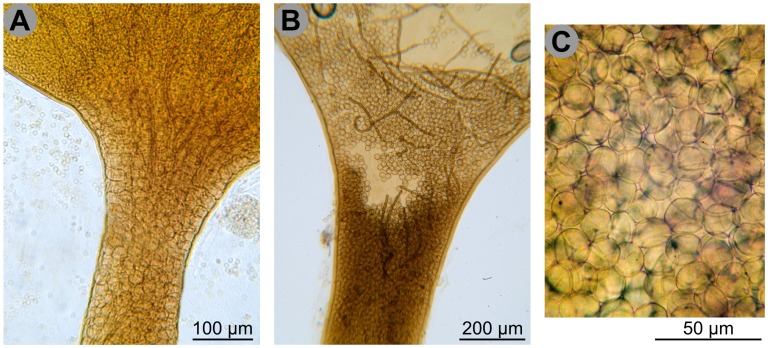
Spore-like bodies. **A.** Vertical section of the stalk and the base of the sporotheca of *Hemitrichia calyculata*, showing the stalk filled with spore-like bodies. Those are larger and clearer than the spores in the sporotheca above, without clear demarcation. **B.** Vertical section of the stalk and the base of the sporotheca of *Trichia decipiens*, showing the stalk filled with spore-like bodies and few capillitial filaments. **C.** Greater magnification of the spore-like bodies of *Trichia decipiens*. Scale and colours are approximate. Credit photos: Michel Poulain.

A previously unrecognised clade associates two *Hemitrichia* species, *H. abietina* and *H. calyculata*, with *Trichia decipiens* (although only well-supported in Bayesian analyses, Fig. 1∶0.99/−, Fig. 2∶1.0/0.59). In spite of their presently being in different genera, there is a striking characteristic shared by these three species: the stalk is filled with “spore-like bodies” (Fig. 7). These structures are formed during sporophore development by cleavage of the cytoplasm: the nuclei in the sporotheca will form spores, the ones in the stalk will become spore-like bodies [45]. The latter are larger than spores, multinucleate and highly vacuolated, and densely packed in the stalk [45] (Fig. 7). Spore-like bodies are characteristic of *Arcyria* and are also found in *Licea operculata* [29]. Establishing a new family for this clade will probably be appropriate but is premature before the type species *Hemitrichia clavata* is investigated, as well as sessile specimens of *Hemitrichia.*

*Arcyria* has mostly stalked sporophores, the peridium disappears but at the base [45], the capillitium is net-forming and never sculptured with spiral bands. The monospecific genus *Arcyodes* is distinguished from *Arcyria* by its thin, shining, persistent peridium and a very short (when present) stalk. *Arcyodes* nests well within *Arcyria*, jointly forming a clear monophyletic clade (1.0/97, Fig. 1). *Arcyodes* is specifically sister to a subset of *Arcyria* species that are pink or red. Our trees suggest that pigmentation may be more fundamental for subdividing this clade (white versus pink/red, Fig. 1) than the differences used to erect *Arcyodes* as a genus. Should this be confirmed by wider sampling, including yellow species, the genus *Arcyodes* may be abandoned.

*Perichaena* is clearly a clade, and is characterized by the combination of generally branched capillitium lacking spiral bands and a thick, persistent peridium. It shares with *Arcyria* and *Arcyodes* the type IV capillitium (one layer, large lumen) (Fig. 2) [40].

*“Trichia* and allied genera” is composed of three subclades in the SSU tree (Fig. 1), with *Trichia varia* as sister of the other two. The “*Trichia* and allied genera” clade is neither robust nor well-supported (0.67/−), but is corroborated by an ultrastructural character of the capillitium: species of *Trichia*, *Metatrichia* and *Oligonema* posses the type 5 (two layers and a narrow lumen) according to Ellis et al. [40] (Fig. 2). Only the *Oligonema*-containing subclade is robust and well-supported (0.98/85, Fig. 1): it includes *Trichia scabra, T. alpina, T. persimilis, Oligonema schweinitzii* and *O. flavidum*. The genus *Oligonema* is characterized by short capillitial threads, similar to those of *Trichia*, but lacking spiral bands (Fig. 6 C). In spite of this difference, the spores of *Oligonema schweinitzii* and *Trichia persimilis* are both reticulate (Fig. 6 C). It has been observed that *Trichia persimilis*, when exposed to severe changes of temperature at the time of fruiting, has produced very short capillitial threads with broad rings and faint spirals with much the same character as *Oligonema schweinitzii*, and some *Trichia* species have developed capillitium with ridges like that of *Cornuvia* [46]. This raises the possibility that *Oligonema* and *Cornuvia* are only aberrant developmental forms of extant *Trichia* species, though it is also possible that mutations could permanently mimc such aberrations in which case they could be genetically distinct. Although a wider phylogeny is needed to corroborate this hypothesis, our results already suggest that *Cornuvia* and *Oligonema* might be unneeded generic names. Since the type species of *Trichia, Trichia varia*, is in a poorly resolved position in the clade, changes appear premature.

Summarizing, no character currently used for higher classification within Lucisporidia is apomorphic, and some monophyletic groups can only be defined by a particular assemblage of few characters: as an example, the new clade 2 is characterized by spore-like bodies in the stalk and capillitium with spiral bands, while *Arcyria* displays spore-like bodies and net-forming capillitium without spiral bands.

### A group I Intron in a Previously Unrecorded Insertion Site

Group I introns are the most abundant self-splicing introns [47], and 119 insertion sites are currently identified in the SSU (The Comparative RNA Web (CRW) Site, http://www.rna.icmb.utexas.edu/SAE/2C/rRNA_Introns/, accessed 17 Sep 2012). Group I introns IC1 have been found in position S1210 in 36 fungal sequences (The Comparative RNA Web (CRW) Site, http://www.rna.ccbb.utexas.edu/SAE/2C/rRNA_Introns/accessed 17 Sep. 2012), but to date never in Myxomycetes, questioning the possible insertion mechanism and origin of the *Licea marginata* S1210. Two pathways are invoked to explain the spread of introns into ectopic sites: the first is reverse splicing of free intron RNA into the target RNA; the second is endonuclease-mediated intron homing, with the homing endonuclease gene inserted in the intron sequence [48], [49]. Currently, none of these pathways can be excluded to explain the Lma.S1210 insertion: the lack of the highly mobile homing endonuclease gene in itself is not conclusive, since it can be easily lost after the intron insertion [48]. Similarly, Lma.S1210 could have been gained by lateral transfer of a fungal S1210, or by ectopic transfer of a SSU intron. Answering these questions could be of general interest to help illustrating group I intron loss and gain.

### Conclusion

Our key findings are that Cribrariidae are deeply divergent from all other Lucisporidia and that the distinction between the orders Liceida and Trichiida is not supported. At the family level, Reticulariidae, Dictydiaethaliidae, Dianemidae and Liceidae (if *Licea variabillis* is excluded) are apparently holophyletic, but Arcyriidae and Trichiidae are jumbled. Several generic or familial boundaries will need revision in future, taking into account a combination of characters, not one character alone. We show the significance of some previously neglected features, like spore-like bodies in the stalk, the capillitium connecting the base of the sporotheca to the peridium, and pigmentation in *Arcyria*. The evolutionary path from individual sporophores to pseudoaethalia and aethalia is confirmed in two independent clades. Ancestral characters are the stalked, individual fruiting bodies arising from a large plasmodium, while small plasmodia giving rise to a single fruiting body are derived.

## Supporting Information

Figure S1 **A**: List of the primers used in this study and their sequences (5′–3′). Colours match the regions in the diagram (B), showing the approximate position of the primers. New primers are in bold, for the others the reference is given. **B:** Schematic diagram of the SSU gene. Numbers indicate corresponding regions in the sequence of *Physarum polycephalum* X13160. Intron insertions positions are indicated by green bars and labels.
(PDF)

Figure S2 EF-1α gene tree of Lucisporidia derived by Bayesian inference of 380 amino-acid positions of 38 taxa, with *Ceratiomyxa fruticulosa* as outgroup. Species names are followed by GenBank accession number, except for sequences obtained during this study (in bold), whose accession numbers and collection sites are in Table S1; Groups are labelled and highlighted as in Fig. 1, with labels in grey if appearing as polyphyletic, in black if monophyletic. Bayesian posterior probabilities (BPP)/ML bootstrap replicates (MLB) are shown for each branch; dashes indicate a conflicting topology in the ML tree; a dot on the line indicates maximum support in both analyses. The scale bar indicates the fraction of substitutions per site.
(PDF)

Figure S3 Schematic secondary structure of the group I intron S1210 found in the SSU sequence of *Licea marginata* JX481296, according to [22]. The putative 5′ and 3′ splice sites (SS) are indicated by an arrow. Flanking exon sequences are in lowercase and outlined. The substrate domains (P1 and P2), the catalytic domains (P3, P7, P8 and P9) and the scaffold domains (P4, P5 and P6) are labelled. When the sequence is not shown, the length of the helix is given.
(PDF)

Table S1 List of new specimens used in this study, GenBank accession numbers and collection information. Herbaria: AMFD = Anna Maria Fiore-Donno, DWM = David Mitchell, HS = Hacène Seraoui, MM = Marianne Meyer, MS = Martin Schnittler.
(PDF)

Table S2 Publicly available sequences not included in this study. **A.** Blast results of the 11 SSU sequences wrongly submitted as Lucisporidia (date: 6 Sep 2012). **B.** List of the EF-1α sequences too short or of poor quality (presence of indels and ambiguities).
(PDF)

Table S3 Length, position and number of introns found in SSU sequences.
(PDF)

Supporting Information S1 SSU alignment in fasta format of the 51 sequences used in Fig. 1. The first sequence indicates the positions retained for phylogenetic analyses.
(FSA)

Supporting Information S2 EF-1α alignment (nucleotides) in fasta format of the 41 sequences used in Fig. S2. The probably obligatory spliceosomal intron starts at position 460. The first sequence indicates the nucleotide positions retained to obtain the amino-acid alignment (Supporting information S3).
(FSA)

Supporting Information S3 EF-1α alignment (amino-acids) in fasta format of the 41 sequences used in Fig. S2.
(FSA)

## References

[1] Stephenson SL, Fiore-Donno AM, Schnittler M (2011) Myxomycetes in soil. Soil Biol Biochem 43: 2237–2242.

[2] Fiore-Donno A-M, Nikolaev SI, Nelson M, Pawlowski J, Cavalier-Smith T, et al (2010) Deep phylogeny and evolution of slime moulds (Mycetozoa). Protist 161: 55–70. 19656720 10.1016/j.protis.2009.05.002

[3] Lahr DJG, Grant J, Nguyen T, Lin JH, Katz LA (2011) Comprehensive phylogenetic reconstruction of Amoebozoa based on concatenated analyses of SSUrDNA and actin genes. PLoS ONE. pp. e22780. 10.1371/journal.pone.0022780 PMC3145751 21829512

[4] Shadwick LL, Spiegel FW, Shadwick JDL, Brown MW, Silberman JD (2009) Eumycetozoa = Amoebozoa?: SSUrDNA phylogeny of protosteloid slime molds and its significance for the amoebozoan supergroup. PLoS ONE. pp. e6754. 10.1371/journal.pone.0006754 PMC2727795 19707546

[5] Hampl V, Hug L, Leigh JW, Dacks JB, Lang FB, et al (2009) Phylogenomic analyses support the monophyly of Excavata and resolve relationships among eukaryotic ‘‘supergroups’’. Proc Natl Acad Sci USA 106: 3859–3864. 19237557 10.1073/pnas.0807880106 PMC2656170

[6] Wegener Parfrey L, Grant J, Tekle YI, Lasek-Nesselquist E, Morrison HG, et al (2010) Broadly sampled multigene analyses yield a well-resolved eukaryotic tree of life. Syst Biol 59: 518–533. 20656852 10.1093/sysbio/syq037 PMC2950834

[7] Tekle YI, Grant J, Anderson RO, Nerad TA, Cole JC, et al (2008) Phylogenetic placement of diverse amoebae inferred from multigene analyses and assesment of clade stability within “Amoebozoa” upon removal of varying rate classes of SSU-rDNA. Mol Phyl Evol 47: 339–352. 10.1016/j.ympev.2007.11.015 18180171

[8] Cavalier-Smith T (2012) Early evolution of eukaryote feeding modes, cell structural diversity, and classification of the protozoan phyla Loukozoa, Sulcozoa, and Choanozoa. Eur J Protistol in press. 10.1016/j.ejop.2012.06.001 23085100

[9] Fiore-Donno A-M, Berney C, Pawlowski J, Baldauf SL (2005) Higher-order phylogeny of plasmodial slime molds (Myxogastria) based on EF1-A and SSU rRNA sequences. J Euk Microbiol 52: 201–210. 15926995 10.1111/j.1550-7408.2005.00032.x

[10] Lado C, Pando F (1997) Myxomycetes, I. Ceratiomyxales, Echinosteliales, Liceales, Trichiales; Real Jardìn Botanìco de Madrid, editor. Madrid, Espana: Cramer, J. 324 p.

[11] Neubert H, Nowotny W, Baumann K (1993) Die Myxomyceten Deutschlands und des angrenzenden Alpenraumes. Band I: Ceratiomyxales, Echinosteliales, Liceales, Trichiales. Gomaringen (D): K. Baumann Verlag. 343 p.

[12] Poulain M, Meyer M, Bozonnet J (2011) Les Myxomycètes; Fédération Mycologique et Botanique Dauphiné-Savoie, editor. Sevrier, France.

[13] Eliasson UH (1977) Recent advances in the taxonomy of Myxomycetes. Bot Notiser 130: 483–492.

[14] Olive LS (1975) The Mycetozoans. New York: Academic Press. 293 p.

[15] Pawlowski J, Adl SM, Audic S, Bass D, Belbahri L, et al.. (2012) CBOL Protist Working Group: Barcoding eukaryotic richness beyond the animal, plant and fungal kingdoms. PloS Biology. pp. e1001419. 10.1371/journal.pbio.1001419 PMC3491025 23139639

[16] Fiore-Donno AM, Kamono A, Meyer M, Schnittler M, Fukui M, et al. (2012) 18S rDNA phylogeny of *Lamproderma* and allied genera (Stemonitales, Myxomycetes, Amoebozoa). PLoS ONE. pp. e35359. 10.1371/journal.pone.0035359 PMC3329430 22530009

[17] Hall TA (1999) BioEdit: a user-friendly biological sequence alignment editor and analysis program for Windows 95/98/NT. Nucleic Acids Symposium Series 41: 95–98.

[18] Posada D (2008) jModelTest: phylogenetic model averaging. Mol Biol Evol 25: 1253–1256. 18397919 10.1093/molbev/msn083

[19] Jobb G (2008) TREEFINDER version of October 2008. Munich, Germany: Available: www.treefinder.de.

[20] Huelsenbeck JP, Ronquist F Mr Bayes v3.0b4. Available: http://morphbankebcuuse/mrbayes/infophp. Accessed 2012 Oct.

[21] Stamatakis A, Ludwig T, Meier H (2005) RAxML-III: A fast program for maximum likelihood-based inference of large phylogenetic trees. Bioinformatics 21: 456–463. 15608047 10.1093/bioinformatics/bti191

[22] Lundblad EW, Einvik C, Ronning S, Haugli K, Johansen S (2004) Twelve group I introns in the same pre-rRNA transcript of the myxomycete *Fuligo septica*: RNA processing and evolution. Mol Biol Evol 21: 1283–1293. 15034133 10.1093/molbev/msh126

[23] Johansen S, Haugen P (2001) A new nomenclature of group I introns in ribosomal DNA. RNA 7: 935–936. 11453066 10.1017/s1355838201010500 PMC1370146

[24] Michel F, Westhof E (1990) Modelling of the three-dimensional architecture of group I catalytic introns based on comparative sequence analysis. J Mol Biol 216: 585–610. 2258934 10.1016/0022-2836(90)90386-Z

[25] Haugen P, Coucheron DH, Ronning SB, Haugli K, Johansen S (2003) The molecular evolution and structural organization of self-splicing group I introns at position 516 in nuclear SSU rDNA of Myxomycetes. J Euk Microbiol 50: 283–292. 15132172 10.1111/j.1550-7408.2003.tb00135.x

[26] Fiore-Donno AM, Novozhilov Y, Meyer M, Schnittler M (2011) Genetic structure of two protist species (Myxogastria, Amoebozoa) suggests asexual reproduction in sexual amoebae. PLoS ONE. pp. e22872. 10.1371/journal.pone.0022872 PMC3148230 21829662

[27] Fiore-Donno AM, Haskins EF, Pawlowski J, Cavalier-Smith T (2009) *Semimorula liquescens* is a modified echinostelid myxomycete (Mycetozoa) Mycologia. 101: 773–776. 10.3852/08-075 19927743

[28] Baldauf SL, Doolittle WF (1997) Origin and evolution of the slime molds (Mycetozoa). Proc Natl Acad Sci USA 94: 12007–12012. 9342353 10.1073/pnas.94.22.12007 PMC23686

[29] Gilert E (1997) Morphological and ultrasctructural features in selected species of *Licea* (Myxomycetes) Nord J Bot. 16: 705–709.

[30] Iwata D, Ishibashi M, Yamamoto Y (2003) Cribrarione B, a new naphthoquinone pigment from the myxomycete *Cribraria cancellata* . J Nat Prod 66: 1611–1612. 14695806 10.1021/np030308e

[31] Harper RA, Dodge BO (1914) The formation of the capillitium in certain Myxomycetes. Ann Bot 28: 1–20.

[32] Kalyanasundaram I (1973) Capillitial development in *Stemonitis*. In: Taxonomy of Fungi I. Subramanian C, editor: University of Madras, India. 9–13.

[33] Mims CW (1969) Capillitial formation in *Arcyria cinerea* . Mycologia 61: 784–798.

[34] Bisby G (1914) Some observations on the formation of the capillitium and the development of *Physarella mirabilis* Peck and *Stemonitis fusca* Roth. Am J Bot 1: 274–288.

[35] Charvat I, Cronshaw J, Ross IK (1974) Development of the capillitium in *Perichaena vermicularis* a plasmodial slime mold. Protoplasma 80: 207–221. 4364220 10.1007/BF01666360

[36] Welden AL (1955) Capillitial development in the Myxomycetes *Badhamia gracilis* and *Didymium iridis* . Mycologia 47: 714–728.

[37] Blackwell M, Alexopoulos CJ (1974) A study of sporophore development in the myxomycete *Protophysarum phloiogenum* . Arch Microbiol 99: 331–344. 4372966 10.1007/BF00696247

[38] Charvat I, Ross IK, Cronshaw J (1973) Ultrastructure of the plasmodial slime mold *Perichaena vermicularis.* II. Formation of the peridium. Protoplasma 78: 1–19. 4796461 10.1007/BF01281519

[39] Hatano T, Keller HW, Arnott HJ (1999) SEM observation on pseudocapillitium of *Lycogala epidendrum* (L.) Fries (Myxomycetes). Hikobia 13: 47–52.

[40] Ellis TT, Scheetz RW, Alexopoulos CJ (1973) Ultrastructural observations on capillitial types in the Trichiales (Myxomycetes). Trans Amer Micros Soc 92: 65–79.

[41] Alexopoulos CJ (1960) Gross morphology of the plasmodium and its possible significance in the relationships among the Myxomycetes. Mycologia 52: 1–20.

[42] Haskins EF, Kerr NS (1978) Vergleich der Plasmodien-Typen und der Sporulation bei Myxomyceten (Film C 1220 des IWF, Göttingen 1976). Publikationen zu Wissenschaftlichen Filmen, Sektion Biologie 11: 1–34.

[43] Clark J, Haskins EF, Stephenson SL (2004) Culture and reproductive systems of 11 species of Mycetozoans. Mycologia 96: 36–40. 21148826

[44] Nannenga-Bremekamp NE (1982) Notes on Myxomycetes. XXI. The use of polarized light as an aid in the taxonomy of the Trichiales. Proc Kon Ned Akad Wetensch, Ser C 85: 541–562.

[45] Mims CW, Rogers MA (1975) A light and electron microscopy study of stalk formation in the Myxomycete *Arcyria cinerea* . Mycologia 67: 638–649.

[46] Lister A (1894) A monograph of the Mycetozoa. London, UK: The British Museum. 224 p.

[47] Cannone JJ, Subramanian S, Schnare MN, Collett JR, D'Souza LM, et al (2002) The Comparative RNA Web (CRW) Site: an online database of comparative sequence and structure information for ribosomal, intron, and other RNAs. BMC Bioinf. 10.1186/1471-2105-3-2 PMC65690 11869452

[48] Haugen P, Simon DM, Bhattacharya D (2005) The natural history of group I introns. Trends Genet 21: 111–119. 15661357 10.1016/j.tig.2004.12.007

[49] Bhattacharya D, Reeb V, Simon DM, Lutzoni F (2005) Phylogenetic analyses suggest reverse splicing spread of group I introns in fungal ribosomal DNA. BMC Evol Biol. 68. 10.1186/1471-2148-5-68 PMC1299323 16300679

