# Supplementary material for: Two-Gene Phylogeny of Bright-Spored Myxomycetes (Slime Moulds, Superorder Lucisporidia)
Source: PLoS One. 2013 May 7;8(5):e62586. doi: 10.1371/journal.pone.0062586 (PMC3646832; doi:10.1371/journal.pone.0062586)

Figure S1. A: List of the primers used in this study and their sequences (5'-3'). Colours match the regions in the diagram (B), showing the approximative position of the primers. New primers are in bold, for the others the reference is given. **B:** Schematic diagram of the SSU gene. Numbers indicate corresponding regions in the sequence of *Physarum polycephalum* X13160. Intron insertions positions are indicated by green bars and labels.

A

	**FORWARD**
S1 [1]	AACCTGGTTGATCCTGCC
**SFATri** [1]	AATCTGCGAACGGCTCCGTA
SF1Tri [2]	CGAACGGCTCCGTATATC
**S4Bright**	TTYGRTCCTGGAGAGGTAGCC
S414F [1]	GAAGGCAGCAGGCGCGCAACG
S4 [1]	AGCAGGCGCGTAACGTTC
**S4T**	TTACCCAATGCTAACACAGCG
**S4Lic**	CAATTAGAGGACAAGTCTGG
718FTri [2]	GTAATTCCAGCTCCAATAGCATCT
718FLic [2]	GTAATTCCAGCTCTAATRGCATC
718FCri [2]	CCGCGGTAATTCCAGCACT
S11Tri [2]	ATCAAGAACGAAAGTCTGGGG
S11Cri [2]	GGGCGAGGGGTGAAATCC
**S11Ret**	GGTAGRGGTGAAATYCGTTGATC
S12Tri [2]	ATCAGATACCATCGTAGTCC
S12Cri [2]	GATTAGATACCGTTGTAGTC
**S12Lyc**	AGATACCACAGTAGTCCAGG
S13 [1]	GAGTATGGTCGCAAGGCTG
S13.5 [1]	GAAACTTAAAGGAATTGACGG
S14Tri [2]	TTGACGGAAGAGCACACAAGG
S14.5 [1]	TAATTTGACTCAACACGGGG
**S14.5Lyc**	TAAATTGACTCAACACGGGG
**S20Ret**	GCCCGTCGCTCCTACCGATT
**S26FTri**	TCAATAACAGGTCAGTGATGCC
S26FCri [2]	TGCAATAACAGGTCAGTGATG
**S28Tri**	CAGTGATGCCCTTAGATGC
**S30Cri**	CGTCGCTCCTACCGATTG

	**REVERSE**
SR4Bright [1]	TGCTGGCACCAGACTTGT
**SR4Lyc**	ACCGGACTTGTCCTCCAGT
**718RTri**	AGATGCTATTGGAGCTGGAATTAC
**753RTri**	CTACGAGCGTTTTAACYGCARCAA
SR11Lic [2]	AAGGATCAACGGATTTCACCC
SR11Cri [2]	GGATTTCACCCCTCGCCC
**SR11Ent**	TCACCTRGCGAGGATCAACG
**SR11Ret**	GATCAACGRATTTCACCYCTACC
SR12Tri [2]	TTAGGGTCTGGACTACGATGG
SR12Lic [2]	CTGGACTACTGTGGTATCTG
SR13 [1]	GGAGTATGGTCGCAAGGCTG
SR13U [3]	TTCAGYCTTGCGRCCATACTCC
SR13Tri [2]	TTCAGTCTTGCGACCATACTCC
SR14Tri [2]	AGGCTCCACTCCTTGTGTG
SR15U [1]	GGTGGTGCATGGCCGTTC
SR18Tri [2]	CACGAACTAAGAACGACCATGC
**SR18Ret**	GCTCGTTADCGGAATTAACCAGAC
S26R [3]	CATCACTGACCTGTTATTGACC
**SR27Lic**	AGCGCTAGCGAGGTTCCC
**SR29Tri**	AGAGCAGGGACAGAATCG
SR30Tri [2]	GGTGTGTACAAAGAGCAGGG
RibTri [2]	TGCAGGTTCACCTACGGATAC

[1] *Published in Fiore-Donno et al. 2008*
[2] *Published in Fiore-Donno et al. 2010*
[3] *Published in Fiore-Donno et al. 2012*

B

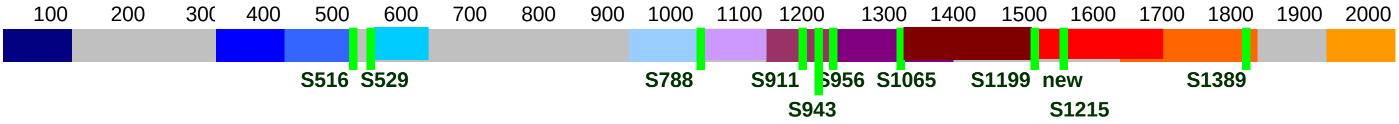

Supplement: Figure S1 — A: List of the primers used in this study and their sequences (5′–3′). Colours match the regions in the diagram (B), showing the approximate position of the primers. New primers are in bold, for the others the reference is given. B: Schematic diagram of the SSU gene. Numbers indicate corresponding regions in the sequence of Physarum polycephalum X13160. Intron insertions positions are indicated by green bars and labels. (PDF) [file pone.0062586.s001.pdf]
