# Supplementary material for: Two-Gene Phylogeny of Bright-Spored Myxomycetes (Slime Moulds, Superorder Lucisporidia)
Source: PLoS One. 2013 May 7;8(5):e62586. doi: 10.1371/journal.pone.0062586 (PMC3646832; doi:10.1371/journal.pone.0062586)
Supplement: Figure S2 — EF-1α gene tree of Lucisporidia derived by Bayesian inference of 380 amino-acid positions of 38 taxa, with Ceratiomyxa fruticulosa as outgroup. Species names are followed by GenBank accession number, except for sequences obtained during this study (in bold), whose accession numbers and collection sites are in Table S1; Groups are labelled and highlighted as in Fig. 1, with labels in grey if appearing as polyphyletic, in black if monophyletic. Bayesian posterior probabilities (BPP)/ML bootstrap replicates (MLB) are shown for each branch; dashes indicate a conflicting topology in the ML tree; a dot on the line indicates maximum support in both analyses. The scale bar indicates the fraction of substitutions per site. (PDF) [file pone.0062586.s002.pdf]

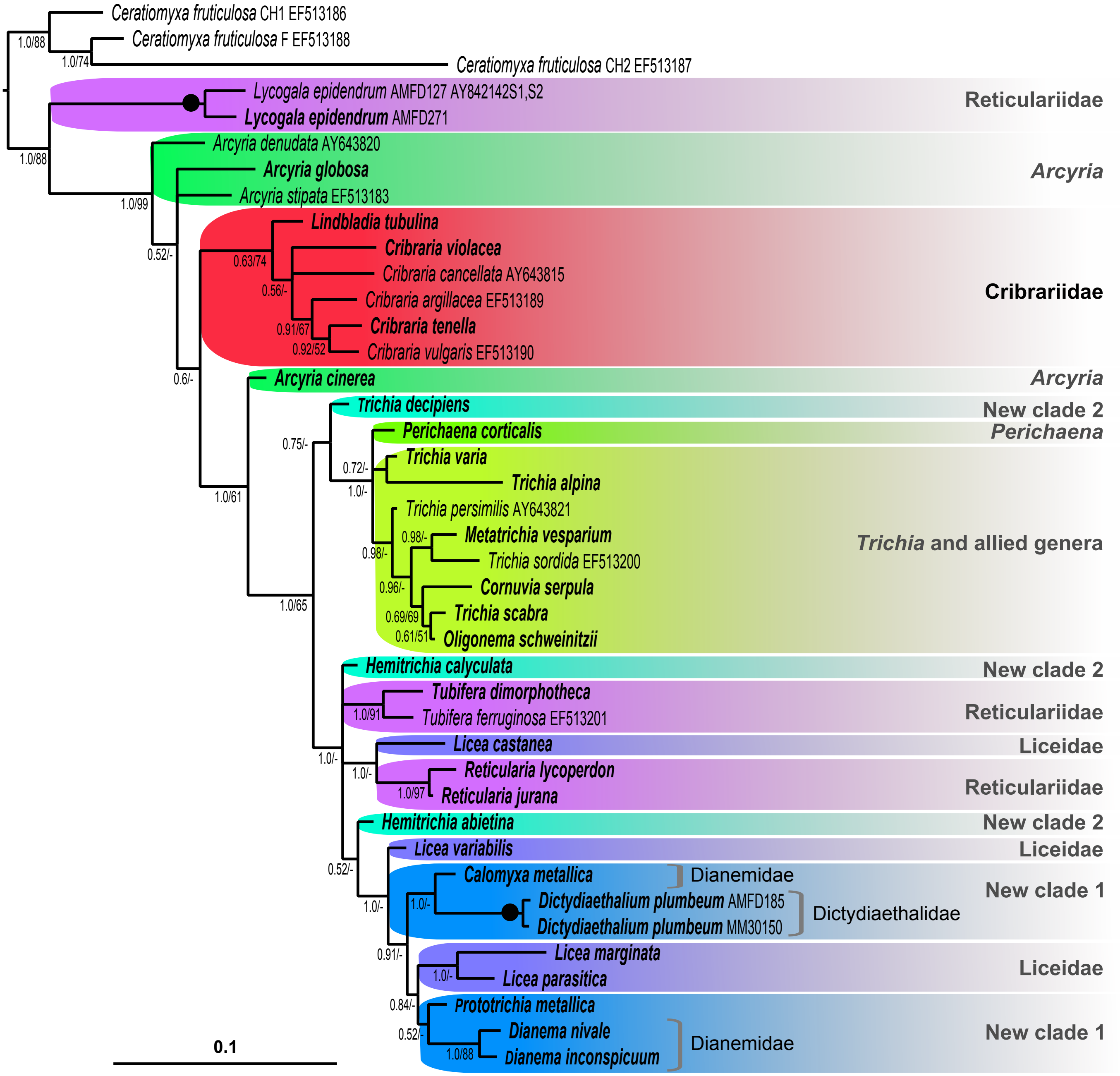

Figure S2. EF-1alpha gene tree of Lucisporidia derived by Bayesian inference of 380 amino-acid positions of 38 taxa, with *Ceratiomyxa fruticulosa* as outgroup. Species names are followed by GenBank accession number, except for sequences obtained during this study (in bold), whose accession numbers and collection sites are in Table S1; Groups are labelled and highlighted as in Fig. 1, with labels in grey if appearing as polyphyletic, in black if monophyletic. Bayesian posterior probabilities (BPP)/ML bootstrap replicates (MLB) are shown for each branch; dashes indicate a conflicting topology in the ML tree; a dot on the line indicates maximum support in both analyses. The scale bar indicates the fraction of substitutions per site.
