# Supplementary material for: Two-Gene Phylogeny of Bright-Spored Myxomycetes (Slime Moulds, Superorder Lucisporidia)
Source: PLoS One. 2013 May 7;8(5):e62586. doi: 10.1371/journal.pone.0062586 (PMC3646832; doi:10.1371/journal.pone.0062586)
Supplement: Figure S3 — Schematic secondary structure of the group I intron S1210 found in the SSU sequence of Licea marginata JX481296, according to [22]. The putative 5′ and 3′ splice sites (SS) are indicated by an arrow. Flanking exon sequences are in lowercase and outlined. The substrate domains (P1 and P2), the catalytic domains (P3, P7, P8 and P9) and the scaffold domains (P4, P5 and P6) are labelled. When the sequence is not shown, the length of the helix is given. (PDF) [file pone.0062586.s003.pdf]

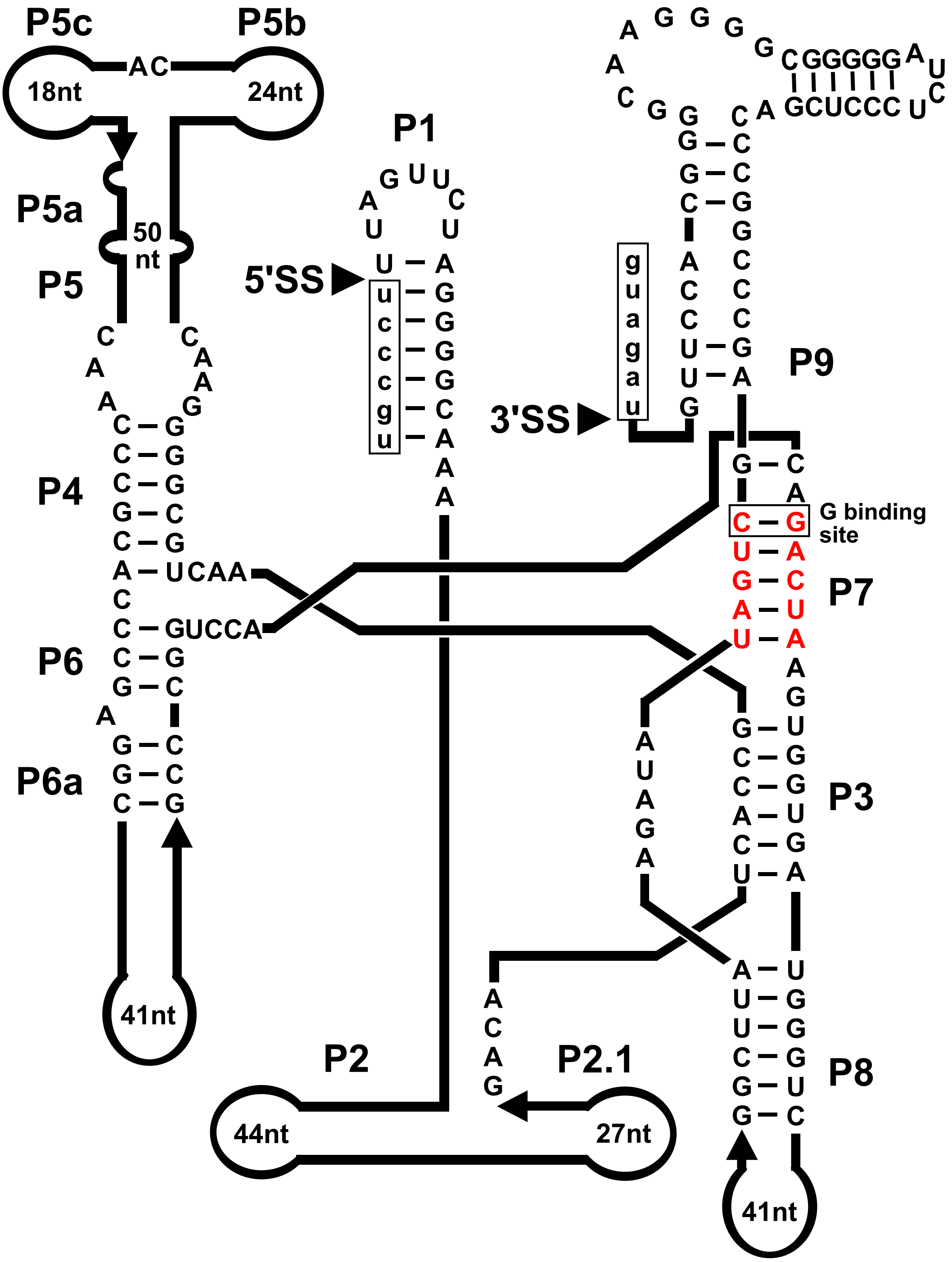

Figure S3. Schematic secondary structure of the group I intron S1210 found in the SSU sequence of *Licea marginata* JX481296, according to (Lundblad et al. 2004). The putative 5' and 3' splice sites (SS) are indicated by an arrow. Flanking exon sequences are in lowercase and outlined. The substrate domains (P1 and P2), the catalytic domains (P3, P7, P8 and P9) and the scaffold domains (P4, P5 and P6) are labelled. When the sequence is not shown, the length of the helix is given.
