# Supplementary material for: Two-Gene Phylogeny of Bright-Spored Myxomycetes (Slime Moulds, Superorder Lucisporidia)
Source: PLoS One. 2013 May 7;8(5):e62586. doi: 10.1371/journal.pone.0062586 (PMC3646832; doi:10.1371/journal.pone.0062586)
Supplement: Table S1 — List of new specimens used in this study, GenBank accession numbers and collection information. Herbaria: AMFD = Anna Maria Fiore-Donno, DWM = David Mitchell, HS = Hacène Seraoui, MM = Marianne Meyer, MS = Martin Schnittler. (PDF) [file pone.0062586.s004.pdf]

Table SI. List of new specimens used in this study, GenBank accession numbers and collection information. Herbaria: AMFD = Anna Maria Fiore-Donno, DWM = David Mitchell, HS = Hacène Seraoui, MM = Marianne Meyer, MS = Martin Schnittler.

Taxon	Authors	Herbarium #	Date	Place of collection	Altitude (m)	Substrate	Lat.	Long.	GenBank accession # SSU	GenBank accession # EF-1alpha
Arcyodes incarnata	(Alb. & Schwein) O.F. Cook	DWM2592	02/09/96	GB, W-Sussex, Billingshurst, Fishers Farm	28	Dead *Fagus*	N 51.0240°	W 0.4834°	JX481280	
Arcyria cinerea	(Bull.) Pers.	AMFD433	06/08/11	DE, Meckl.-Vorpommen, Greifswald	8	Decayed broad-leaved log	N 54.0824°	E 13.4468°	JX481281	JX481317
Arcyria globosa	Schwein.	AMFD252	17/08/05	MX, Hidalgo, Tlanchinol	1300	Dead leaves	N 21.3667°	W 98.050°	JX481282	JX481318
Arcyria marginoundulata	Nann.-Bremek. & Y.Yamam.	MM37736	27/12/08	TH, rambutan bought in a French shop	n/a	Rambutan peel	n/a	n/a	JX481283	
Calomyxa metallica	(Berk.) Nieuwl.	AMFD483	20/09/08	GB, Oxfordshire, Oxford	56	Living *Sambucus nigra* bark	N 51.7642°	W 01.2808°	JX481284	JX481319
Cornuvia serpula	(Wigand) Rost.	MM29198	03/04/04	FR, Var, Port-Cros	10	*Quercus ilex* litter	N 43.0067°	E 06.3975°	JX481285	JX481320
Cribraria tenella	Schrad.	AMFD148	10/06/04	CH, Geneva, Céligny	450	Decayed log	N 46.3651°	E 06.1856°	JX481286	JX481321
Cribraria violacea	Rex	AMFD172	03/10/04	IT, Puglia, Lecce	20	*Cupressus sempervirens* bark	N 40.3822°	E 18.2601°	JX481287	JX481322
Dianema inconspicuum	Poulain, Meyer & Bozonnet	MM24067	11/06/04	FR, Savoy, La Bathie	1804	*Vaccinium myrtillus* twigs	N 45.6503°	E 06.4794°	JX481288	JX481323
Dianema nivale	(Meyl.) G.Lister	MM 29888	18/06/06	FR, Savoy, Col de la Madeleine	1988	Dead branches	N 45.4389°	N 06.3775°	JX481289	JX481324
Dictydiaethalium dictyosporum	Nann.-Bremek.	HS3379	04/10/08	NC, Numea, Buluparis	n/a	Log	S 21.8360°	E 166.1665°	JX481290	
Dictydiaethalium plumbeum AMFD185	(Schum.) Rostaf.	AMFD185	06/11/04	CH, Geneva, Céligny	450	Broad-leaved stump	N 46.3651°	E 06.1856°	JX481291	JX481325
Dictydiaethalium plumbeum MM30150	(Schum.) Rostaf.	MM30150	27/11/06	FR, Savoy, Saint-Paul s/Isère	477	Broad-leaved branch	N 45.6056°	E 06.4406°	JX481292	JX481326
Hemitrichia abietina	(Wigand) G.Lister	AMFD213	06/05/05	FR, Savoy, Essert-Blay	1650	Log	N 45.6197°	E 06.3962°	JX481293	JX481327
Hemitrichia calyculata	(Speg.) M.L.Farr	MS 22060	22/10/10	DE, Meckl.-Vorpommen, Greifswald	10	*Alnus glutinosa* decayed log	N 54.1278°	E 13.3456°	JX481294	JX481328
Licea castanea	G. Lister	AMFD102	13/11/02	GB, Yorkshire, Allerthorpe	24	*Pinus* decayed log	N 53.9198°	W 0.8497°	JX481295	JX481329
Licea marginata	Nann.-Bremek.	DWM7368	05/10/08	GB, East Kent, Faversham	36	*Quercus robur* bark	N 51.3027°	E 00.8317°	JX481296	JX481330

Licea parasitica	(Zukal) G.W.Martin	AMFD341	03/11/08	GB, East Kent, Faversham	36	*Quercus robur* bark	N 51.3027°	E 00.8317°	JX481297	JX481331
Licea variabilis	Schrad.	MM28571	15/09/02	FR, Isère, Engins	967	Dead, standing *Pinus*	N 45.1908°	E 05.6177°	JX481298	JX481332
Lindbladia tubulina	Fr.	AMFD228	11/08/05	MX, Tlaxcala, Tlaxco	2956	Log	N 19.66498°	W 98.0854°	JX481299	JX481333
Lycogala epidendrum AMFD127	(L) Fries	AMFD127	25/10/03	CH, Vaud, Allaman	400	*Pinus* log	N 46.4671°	E 06.4108°	JX481300	
Lycogala epidendrum AMFD271	(L) Fries	AMFD271	30/09/07	DE, Saxony, Pirna	249	Decayed log	N 50.9772°	E 14.0389°	JX481301	JX481334
Metatrichia floriformis	(Schwein.) Nann.-Bremek.	MS24827	28/09/10	DE, Thüringen, Mihla	400	Decayed *Fagus* log	N 51.0886°	E 10.3767°	JX481302	
Metatrichia vesparium	(Batsch) Nann.-Bremek. ex G.W. Martin & Alexop.	DWM7019	21/09/05	GB, East Sussex. Ardingly	141	*Betula*	N 51.0676°	W 0.0878°	JX481303	JX481335
Oligonema flavidum	(Peck) Peck	DWM5764	14/08/99	US, Missouri, Mingo reserve	126	Decayed log	N 36.9181°	W 90.3694°	JX481304	
Oligonema schweinitzii	(Berk.) Martin	MM29842	04/05/06	MA, Kenitra, Mamora Forest	40	*Quercus suber* log	N 34.2408°	W 06.5654°	JX481305	JX481336
Perichaena corticalis	(Batsch.) Rost.	AMFD157	15/11/03	CH, Geneva, Chancy	392	*Populus* log	N 46.1358°	E 05.9695°	JX481306	JX481337
Perichaena depressa	Lib.	AMFD256	02/03/05	CH, Geneva, Pt-Lancy	411	Living *Malus* bark	N 46.1927°	E 6.1243°	JX481307	
Perichaena luteola	(Kowalski) Gilert	DWM4984	22/11/93	ES, Isla de la Palma	n/a	Mule dung	n/a	n/a	JX481308	
Prototrichia metallica	(Berk.)Massee	MM24907	06/05/05	FR, Savoy, Essert-Blay	1170	Twig	N 45.6142°	E 06.4139°	JX481309	JX481338
Reticularia jurana	Meylan	AMFD290	25/08/07	GB, Kent, Lamberhurst	76	Broad-leaved log	N 51.0936°	E 0.4038°	JX481310	JX481339
Reticularia lycoperdon	Bull.	AMFD262	11/04/07	GB, Yorkshire, York	30	Window frame	N 53.9627°	W 01.0788°	JX481311	JX481340
Trichia alpina	Meylan	AMFD64	25/05/01	FR, Savoy, Col de Saisie	1620	Living twig	N 45.7453°	E 06.539°	JX481312	JX481341
Trichia decipiens	(Pers.)Macbr.	AMFD159	15/11/03	CH, Geneva, Chancy	392	*Populus* log	N 46.1358°	E 05.9695°	JX481313	JX481342
Trichia scabra	Rostaf.	MS22055	17/11/10	DE, Meckl.-Vorpommen, Neuenkirche	10	Decayed *Betula* log	N 54.1256°	E 13.3494°	JX481314	JX481343
Trichia varia	(Pers.) Pers.	AMFD451	04/10/11	DE, Saxony, Grosser Zschand	264	Decayed log	N 50.8997°	E 14.2961°	JX481315	JX481344
Tubifera dimorphotheca	Nann.-Bremek. & Loer.	AMFD251	17/08/05	MX, Hidalgo, Tlanchinol	1336	Decayed stump	N 20.9843°	W 98.6309°	JX481316	JX481345
