# Supplementary material for: Two-Gene Phylogeny of Bright-Spored Myxomycetes (Slime Moulds, Superorder Lucisporidia)
Source: PLoS One. 2013 May 7;8(5):e62586. doi: 10.1371/journal.pone.0062586 (PMC3646832; doi:10.1371/journal.pone.0062586)
Supplement: Table S2 — Publicly available sequences not included in this study. A. Blast results of the 11 SSU sequences wrongly submitted as Lucisporidia (date: 6 Sep 2012). B. List of the EF-1α sequences too short or of poor quality (presence of indels and ambiguities). (PDF) [file pone.0062586.s005.pdf]

Table S2. **Publicly available sequences not included in this study**. **A.** Blast results of the 11 SSU sequences wrongly submitted as Lucisporidia (date: 6 Sep 2012). **B.** List of the EF-1alpha sequences too short or of poor quality (presence of indels and ambiguities).

A

GenBank Accession #	Organism name	Blast best hit	E-Value	% Identity	% Coverage	Belongs to (higher taxon)
AY145523	*Arcyria cinerea*	*Mattesia geminata* AY334568	0.0	79	100	Alveolata, Apicomplexa
HM101143	*Arcyria nigella*	*Taphrina johansonii* AJ495835	2e-47	85	21 (chimera?)	Fungi, Ascomycetes
JQ812659	*Dianema subretisporum*	*Diderma meyerae* JQ812626	0.0	100	100	Myxomycetes, Fuscisporida
AY237160	*Hemitrichia clavata*	*Corydalis saxicola* AY640053	0.0	84	100	Plant, Papaverales
AY223841	*Hemitrichia serpula*	*Corydalis saxicola* AY640053	3e-95	81	89	Plant, Papaverales
AY145525	*Lycogala flavofuscum*	*Cordyceps cicadae* DQ838788	0.0	99	100	Fungi, Ascomycetes
AY187083	*Lycogala flavofuscum*	*Sporobolomyces folliicola* AB021671	0.0	99	100	Fungi, Basidiomycetes
DQ459629	*Lycogala flavofuscum*	*Sporobolomyces folliicola* AB021671	0.0	99	100	Fungi, Basidiomycetes
JQ277925	*Lycogala flavofuscum*	*Nectriopsis violacea* AY489687	0.0	97	97	Fungi, Ascomycetes
JX273061	*Lycogala sp.*	Uncultured fungus clone AB534515	0.0	99	100	Fungi
AF542044	*Metatrichia vesparium*	*Corydalis saxicola* AY640053	0.0	89	92	Plant, Papaverales
AY187084	*Trichia scabra*	*Aspergillus penicillioides* AB003077	0.0	100	99	Fungi, Ascomycetes

B

		Length (nucleotides)	Indels	Ambiguities	Remarks
FJ546658	*Arcyria cinerea*	616	1	2	Indel disrupting the reading frame
FJ546659	*Arcyria denudata*	707		5	Too many ambiguities
JQ277912	*Arcyria obvelata*	365			Unverified by GenBank staff
JF339220	*Arcyria oerstedii*	329			Unverified by GenBank staff
FJ546665	*Cribraria cancellata* var. *fusca*	540		1	Last 16 bases not alignable
FJ546672	*Hemitrichia calyculata*	709	1	7	First 90 bases not alignable
FJ546676	*Hemitrichia clavata*	600		1	Too short
JQ277910	*Hemitrichia clavata*	362			Unverified by GenBank staff
JF263589	*Lycogala conicum*	570			Unverified by GenBank staff
FJ546677	*Lycogala epidendrum*	719	2	1	Indel disrupting the reading frame
JF263590	*Lycogala flavofuscum*	546			Unverified by GenBank staff
FJ546678	*Metatrichia vesparia* (sic)	1066	3	8	Indel disrupting the reading frame
JQ277908	*Metatrichia vesparium*	362			Unverified by GenBank staff
FJ546690	*Trichia alpina*	735		2	Too short
FJ546691	*Trichia decipiens*	754		1	Too short
FJ546692	*Trichia persimilis*	812	3	0	Indel disrupting the reading frame
FJ546673	*Trichia varia*	736	1	1	Indel disrupting the reading frame
FJ546674	*Trichia verrucosa*	700	1	8	Indel disrupting the reading frame
FJ546675	*Tubulifera arachnoidea* (=*Tubifera ferruginosa*)	721	2	1	Indel disrupting the reading frame
