# Supplementary material for: Two-Gene Phylogeny of Bright-Spored Myxomycetes (Slime Moulds, Superorder Lucisporidia)
Source: PLoS One. 2013 May 7;8(5):e62586. doi: 10.1371/journal.pone.0062586 (PMC3646832; doi:10.1371/journal.pone.0062586)
Supplement: Table S3 — Length, position and number of introns found in SSU sequences. (PDF) [file pone.0062586.s006.pdf]

**Table S3**. Length, position and number of the 37 introns found in the SSU sequences.

| Taxon | Introns names and length (nucleotides) | | | | | | | | | | Total length: | | | Introns | |
|---|---|---|---|---|---|---|---|---|---|---|---|---|---|---|---|
| | **S516** | **S529** | **S788** | **S911** | **S943** | **S956** | **S1065** | **S1199** | **new** | **S1389** | **exon** | **introns** | **Total** | **#** | **%** |
| *Arcyodes incarnata* | | | | | | | | | | | 2027 | | 2027 | | |
| *Arcyria cinerea* | | | | | | | | | | | 1863 | | 1863 | | |
| *Arcyria globosa* | 459 | | | | | | | | | | 1785 | 459 | 2244 | 1 | 20 |
| *Arcyria marginoundulata* | | | | | | | | | | | 1788 | | 1788 | | |
| *Calomyxa metallica* | | | | 662 | | | | | | 533 | 2169 | 1195 | 3364 | 2 | 36 |
| *Cornuvia serpula* | | | | | | | | | | | 1736 | | 1736 | | |
| *Cribraria tenella* | | | | | | | | | | | 1417 | | 1417 | | |
| *Cribraria violacea* | | | | | | 1111 | | | | | 2060 | 1111 | 3171 | 1 | 35 |
| *Dianema inconspicuum* | | | | | | | | | | | 1501 | | 1501 | | |
| *Dianema nivale* | 538 | | | | | | | | | | 2201 | 538 | 2739 | 1 | 20 |
| *Dictydiaethalium dictyosporum* | | | | | | | | | | | 1801 | | 1801 | | |
| *Dictydiaethalium plumbeum* AMFD185 | | | | | | | | | | | 1760 | | 1760 | | |
| *Dictydiaethalium plumbeum* MM30150 | | | | | | 1237 | | | | | 1763 | 1237 | 3000 | 1 | 41 |
| *Hemitrichia abietina* | | | | | | 413 | | | | | 1663 | 413 | 2076 | 1 | 20 |
| *Hemitrichia calyculata* | | | | | | | | | | | 1203 | | 1203 | | |
| *Licea castanea* | | | | | | | | | | | 1486 | | 1486 | | |
| *Licea marginata* | | 399 | 432 | 367 | | | 528 | 349 | 397 | 455 | 1893 | 2927 | 4820 | 7 | 61 |
| *Licea parasitica* | 1149 | | 1010 | 857 | 298 | | | | | | 1439 | 3314 | 4753 | 4 | 70 |
| *Licea variabilis* | | | | | | | | | | | 2116 | | 2116 | | |
| *Lindbladia tubulina* | | | | | | 908 | | | | | 2352 | 908 | 3260 | 1 | 28 |
| *Lycogala epidendrum* AMFD127 | | 722 | | 416 | | | | | | | 2678 | 1138 | 3816 | 2 | 30 |
| *Lycogala epidendrum* AMFD271 | | 778 | | | | 780 | | | | | 2888 | 1558 | 4446 | 2 | 35 |
| *Metatrichia floriformis* | | 1060 | | | | 514 | | | | | 1791 | 1574 | 3365 | 2 | 47 |
| *Metatrichia vesparium* | | | | | | | | | | | 1941 | | 1941 | | |
| *Oligonema flavidum* | 663 | | | | | | | | | | 1860 | 663 | 2523 | 1 | 26 |
| *Oligonema schweinitzii* | 1229 | | | 510 | | | | 533 | | | 1690 | 2272 | 3962 | 3 | 57 |
| *Perichaena corticalis* | | | | | | | | | | | 1869 | | 1869 | | |
| *Perichaena depressa* | | | | | | | | | | | 1768 | | 1768 | | |
| *Perichaena luteola* | | | | | | | | | | | 1872 | | 1872 | | |
| *Prototrichia metallica* | | | | | | | | | | | 2232 | | 2232 | | |
| *Reticularia jurana* | 996 | 478 | | | | | | | | | 717 | 1474 | 2191 | 2 | 67 |
| *Reticularia lycoperdon* | 494 | | | | | | | | | | 877 | 494 | 1371 | 1 | 36 |
| *Trichia alpina* | | | | | | | | | | | 1831 | | 1831 | | |
| *Trichia decipiens* | | | | | | | | | | | 1906 | | 1906 | | |
| *Trichia scabra* | | | | | | | | | | | 1733 | | 1733 | | |
| *Trichia varia* | 443 | | | 507 | | 450 | | 531 | | | 1934 | 1931 | 3865 | 4 | 50 |
| *Tubifera dimorphotheca* | | | | | | 1557 | | | | | 2349 | 1557 | 3906 | 1 | 40 |
| **Total introns/position** | **8** | **5** | **2** | **6** | **1** | **8** | **1** | **3** | **1** | **2** | | | | | |
